# Vitamin D and gene networks in human osteoblasts

**DOI:** 10.3389/fphys.2014.00137

**Published:** 2014-04-09

**Authors:** Jeroen van de Peppel, Johannes P. T. M. van Leeuwen

**Affiliations:** Department of Internal Medicine, Bone and Calcium MetabolismErasmus MC, Rotterdam, Netherlands

**Keywords:** vitamin D, osteoblast, differentiation, mineralization, autocrine/paracrine mechanisms, immune system

## Abstract

Bone formation is indirectly influenced by 1,25-dihydroxyvitamin D3 (1,25D3) through the stimulation of calcium uptake in the intestine and re-absorption in the kidneys. Direct effects on osteoblasts and bone formation have also been established. The vitamin D receptor (VDR) is expressed in osteoblasts and 1,25D3 modifies gene expression of various osteoblast differentiation and mineralization-related genes, such as alkaline phosphatase (ALPL), osteocalcin (BGLAP), and osteopontin (SPP1). 1,25D3 is known to stimulate mineralization of human osteoblasts *in vitro*, and recently it was shown that 1,25D3 induces mineralization via effects in the period preceding mineralization during the pre-mineralization period. For a full understanding of the action of 1,25D3 in osteoblasts it is important to get an integrated network view of the 1,25D3-regulated genes during osteoblast differentiation and mineralization. The current data will be presented and discussed alluding to future studies to fully delineate the 1,25D3 action in osteoblast. Describing and understanding the vitamin D regulatory networks and identifying the dominant players in these networks may help develop novel (personalized) vitamin D-based treatments. The following topics will be discussed in this overview: (1) Bone metabolism and osteoblasts, (2) Vitamin D, bone metabolism and osteoblast function, (3) Vitamin D induced transcriptional networks in the context of osteoblast differentiation and bone formation.

## Bone metabolism and osteoblasts

Bone is formed during fetal development by two processes: endochondral and intramembranous ossification (for review Bilezikian et al., 2002). Skull and flat bones are formed by intramembranous ossification where there is direct bone formation by condensation of the mesenchyme without a preformed cartilaginous scaffold. Long bones and most of the remaining bones are formed by endochondral ossification (Mackie et al., 2008, 2011; Nishimura et al., 2012). This type of bone formation is characterized by the transition of cartilage into mineralized bone tissue.

Two major processes occur in bone: bone modeling and bone remodeling. While bone modeling drives the growth of the skeleton, bone remodeling is responsible for the maintenance of healthy bone in the adulthood (Teti, 2011). Bone remodeling takes place throughout live and maintains the structural integrity and strength of the bone by removing old or damaged bone and replacing it by new, strong bone. Remodeling is a local process that can take place anywhere on the bone surface throughout the lifespan of a bone. Remodeling occurs in a temporary anatomic unit of osteoclasts and osteoblasts called a bone multicellular unit (BMU) (Martinello et al., 2012; Sims and Martin, 2014). The BMU is a sealed compartment in which bone resorption and subsequent formation are regulated. This coupled resorption and formation characterizes and differentiates bone remodeling from bone modeling, in which bone resorption and formation do not have to occur at the same time and site. For growth and for the maintenance of healthy bone, multiple cell types are of importance: mesenchymal stem cells (MSC), osteoblasts, osteocytes, and hematopoietic stem cells and osteoclasts. The osteoblasts play a pivotal role in bone metabolism by forming bone but also by controlling and regulating the formation and activity of the bone resorbing cell the osteoclast.

Osteoblasts originate from MSC. MSCs are located in the bone marrow but also in almost all other tissues undergoing continuous tissue homeostasis. MSCs can differentiate into osteoblasts, chondrocytes, fibroblasts, adipocytes or myocytes (Friedenstein et al., 1974; Minguell et al., 2001; Yin, 2006). During osteoblast differentiation several functional phases can be identified: proliferation, production and maturation of extracellular matrix (ECM) and ECM mineralization (Owen et al., 1991). Osteoblast differentiation can start by a trigger of certain growth factors (Wang, 1993) as well as hormones and other factors (Eijken et al., 2006). Mature osteoblasts produce and secrete ECM molecules (Owen et al., 1991). Osteoblasts synthesize the most abundant bone ECM protein collagen type I but also a broad range of non-collagenous ECM proteins. Mineralization of the ECM is likely induced by matrix vesicles which derive from osteoblasts (Anderson et al., 2005). When mature osteoblasts initiate mineralization of mature ECM, its fate may vary. Osteoblasts can further differentiate into osteocytes, become a bone lining cell or undergo apoptosis (Jilka et al., 1998; Weinstein et al., 1998). Osteoblasts become osteocytes by being entrapped in self-produced ECM, in which they may survive for decades. Osteocytes form a star-shaped network of cytoplasmic extensions. Osteocytes are thought to function as orchestrators of bone by sensing and communicating mechanical stress (i.e., bone damage) via these extensions (Bonewald, 2011). It has become evident by genetic approaches that osteocytes play a role in regulation of bone turn-over (resorption and formation) (Nakashima et al., 2011; Atkins and Findlay, 2012). Bone lining cells are less well understood. They are covering the bone surface and prevent it from being in direct contact with the bone marrow. It has been reported that these cells “clean” resorption pits after osteoclasts retreated (Everts et al., 2002). Bone lining cells are considered as inactive osteoblasts. It has been suggested that these cells can be activated to become osteoblasts (Dobnig and Turner, 1995; Chow et al., 1998) but they also may represent the osteoblastic part of the stem cell niche and interact with the hematopoietic stem cells.

The osteoblasts/osteocytes guarantee the close coupling between bone formation and resorption in healthy bone remodeling. Osteoblasts and osteocytes produce the soluble osteoclast stimulating factors RANKL and M-CSF which upon binding to its receptors (RANK and c-Fms, respectively) induce differentiation of osteoclast progenitors and fusion of mononuclear cells into multinucleated tartrate-resistant acid phosphatase positive osteoclasts (Boyle et al., 2003). Besides RANKL, osteoblasts also produce a soluble decoy-receptor: osteoprotegerin (OPG). OPG binds RANKL with high affinity leading to inhibition of osteoclast stimulation and thus leading to less bone resorption (Lacey et al., 1998; Kostenuik and Shalhoub, 2001).

## Vitamin D, bone metabolism, and osteoblast

The biologically most active form of vitamin D, 1α,25-dihydroxyvitamin D3 [calcitriol or 1,25(OH)2D3 (1,25D3)] is formed by a stepwise process starting in the skin and involving the liver and kidney. Upon ultraviolet B exposure, 7-dehydrocholesterol (pro-vitamin D3) is transformed into (pre)vitamin D3 (cholecalciferol) in the skin. Subsequent hydroxylation at the C25 and 1α position in liver and kidney, respectively, produce 1,25D3 (Holick, 1995). In bone diseases, vitamin D is used as an anti-rickets agent (Kitanaka et al., 1998; McCollum et al., 2002; Tatsumi et al., 2007), which improves bone mineralization and is often prescribed in combination with other osteoporosis drugs to secure a positive calcium balance. However, a recent metaanalyes by Reid et al suggests that the supplementation of vitamin D for the prevention of osteoporosis is inappropriate without specific risk factors for vitamin D deficiency (Reid et al., 2014).

Up to today it is still in debate whether 1,25D3 effects on bone formation are indirect via intestinal and renal regulation of calcium levels or also via a direct effect on osteoblasts. It has been demonstrated that mice lacking the vitamin D receptor (VDR) gene display retarded growth, severe bone impairment, immune abnormalities, and premature death at only 15 weeks of age due to hypocalcemia (Li et al., 1997; Yoshizawa et al., 1997; Mathieu et al., 2001). A rescue diet restored all pathological effects suggesting that as long as calcium homeostasis is under control, bone itself does not seem to be affected by impaired VDR signaling. The importance of physiological 1,25D3 levels for bone is demonstrated by the mutation of the CYP27B1 gene. Subjects with a mutation in that gene develop vitamin-D-dependent rickets (Li et al., 1997). In a mouse model for rickets, greater extensibility and lower stiffness of fibrils resulted from a decreased grade of mineral deposition (Karunaratne et al., 2012). This further supports the importance of an optimal grade of mineralization for healthy bones (Kitanaka et al., 1998) and points to a role for 1,25D3 herein.

A direct positive effect an 1,25D3 analog on bone formation in ovariectomized rats with only slight changes in serum calcium points to the existence of a direct effect on bone formation (Shevde et al., 2002). This is supported by *in vitro* studies demonstrating direct effects on osteoblasts. The VDR is present in osteoblasts and its expression can be regulated by 1,25D3 itself and by other factors such as parathyroid hormone (PTH), glucocorticoids, transforming growth factor-β, and epidermal growth factor (Pols et al., 1988a,b; Reinhardt and Horst, 1990; van Leeuwen et al., 1991, 1992a,b; Godschalk et al., 1992). The expression of VDR allows 1,25D3 to directly affect osteoblast growth and differentiation. 1,25D3 has been shown to stimulate bone formation and mineralization in all studies using human osteoblasts and stimulate osteogenic differentiation from human mesenchymal stem/stromal cells (MSC) (Ueno et al., 1992; Prince et al., 2001; Jørgensen et al., 2004; Van Driel et al., 2006a,b; Zhou et al., 2006, 2012). 1,25D3 enhanced mineralization by effects on human osteoblasts prior to the onset of mineralization (Woeckel et al., 2010). Thus, 1,25D3 is not directly involved in the process of mineral deposition but more likely in a process preparing the environment/ECM for mineralization. 1,25D3 regulates the osteoblast differentiation marker ALPL and various bone ECM proteins such as COL1A1. Procollagen type I by human osteoblasts was stimulated (Franceschi et al., 1988; Hicok et al., 1998) as well as unaffected (Ingram et al., 1994; Hicok et al., 1998; Siggelkow et al., 1999) by vitamin D. However, gene expression profiling studies demonstrated that the 1,25D3 effect in the pre-mineralization phase is not likely primarily due to changes in expression of ECM proteins and thereby composition of the ECM (Woeckel et al., 2010). Production of alkaline phosphatase (ALPL) positive matrix vesicles was significantly induced by 1,25D3 in this period of osteoblast differentiation (Anderson, 1995) providing a means to enhance mineralization (Woeckel et al., 2010). In addition, previous studies have shown the importance of other factors like TGFβ, IGF-I, bone morphogenetic protein, interferon, PTH, hepatocyte growth factor, epidermal growth factor, and peroxisome proliferator-activated receptor ligands and Wnt signaling for the eventual effect of 1,25D3 on osteoblasts (Petkovich et al., 1987; Pols et al., 1988b; Scharla et al., 1991; Bonewald et al., 1992; Godschalk et al., 1992; van Leeuwen et al., 1992a,b; Ingram et al., 1994; Staal et al., 1994, 1996, 1998; Haussler et al., 1998; Yanagisawa et al., 1999; Sammons et al., 2004; Yarram et al., 2004; Fretz et al., 2007; Chen et al., 2012a, 2013; Woeckel et al., 2012; Yamaguchi and Weitzmann, 2012). These data stress the importance of studying and interpreting the effects of 1,25D3 on bone in a systems biological approach encompassing the different layers of regulation and interactions.

In contrast to human and rat studies, 1,25D3 inhibits differentiation and mineralization in cultures of murine osteoblasts (Shi et al., 2007; Chen et al., 2012a,b, 2013) and murine VDR deficient osteoblasts have increased osteogenic potential (Sooy et al., 2004). 1,25D3 increases in a VDR-dependent manner the expression of progressive ankylosis (ANK) and ectonucleotide pyrophosphatase phosphodiesterase (ENPP1) in murine osteoblasts. This leads to an increase in the level of pyrophosphate (PPi) that inhibits mineralization (Lieben et al., 2012). 1,25D3 also increases osteopontin shown to inhibit mineralization (Staal et al., 1996). However, transgenic murine models with osteoblast-specific VDR overexpression show increased bone formation and mineralization (Gardiner et al., 2000; Misof et al., 2003; Xue et al., 2006). An 1,25D3 analog had a positive effect on bone nodule formation and mineralization in murine calvarial osteoblast cultures of wild type but not VDR null mice (Shevde et al., 2002) while one study showed increased mineralization in MC3T3 cell cultures (Matsumoto et al., 1991). In a recent study, Yamamoto et al. (2013) illustrated that mice lacking VDR in osteoblasts had an increased bone mass, due to decreased bone resorption.

Overall the present data show variation in effects of 1,25D3 on differentiation and mineralization with overall stimulatory effects in human and rat osteoblasts while overall an inhibitory effect in murine osteoblasts (Van Driel et al., 2006a). Following this, 1,25D3 has been shown to increase RUNX2 expression in human osteoblasts (Prince et al., 2001; Viereck et al., 2002; Maehata et al., 2006) while 1,25D3 suppresses RUNX2 promoter and reduces RUNX2 expression in murine osteoblasts (Prince et al., 2001; Drissi et al., 2002). Osteocalcin (BGLAP) is an interesting gene considering differences in 1,25D3 effects in human and murine osteoblasts (Thomas, 2000). 1,25D3 stimulates BGLAP expression in human and rat osteoblasts while it inhibits BGLAP expression in murine osteoblasts (Lian et al., 1997; Zhang et al., 1997), supporting differences between human/rat osteoblasts and murine with respect to 1,25D3 responsiveness and mineralization.

A full explanation for this apparent discrepancy between human and murine osteoblasts is absent. Both the extracellular milieu (i.e., presence/absence of growth factors, cytokines and other signaling molecules) and the intracellular milieu (e.g., the insulin-like growth factor binding protein-6 that can bind to the VDR and inhibit 1,25D3 induction of ALPL activity) of the cell is important for the eventual effect of 1,25D3 (Cui et al., 2011). Also the extracellular phosphate concentration may affect the 1,25D3 action (Ito et al., 2013). These characteristics may contribute to the differences in 1,25D3 effects observed in human and murine osteoblasts.

Besides stimulation of bone formation /mineralization by osteoblasts 1,25D3 has certain protective control mechanisms in place to avoid pathological over-mineralization. For example, 1,25D3 induces BGLAP and SPP1, established inhibitors of mineralization (Noda et al., 1990; MacDonald et al., 1993) and a stimulator of mineralization, bone sialoprotein (IBSP), is inhibited by 1,25D3 (Li and Sodek, 1993). As mentioned above also the presence or absence of other growth factors, cytokines or signaling molecules may limit the 1,25D3 effect. Examples of this in relation to mineralization are Activin A and follistatin. Activin A inhibits osteoblast differentiation and mineralization (Eijken et al., 2007). Activin A expression in human osteoblasts is stimulated by 1,25D3 (Woeckel et al., 2013), implicating that 1,25D3 as stimulator of human osteoblast differentiation and mineralization also stimulates the production of a mineralization inhibitor. A function in the prevention of over-mineralization is supported by the data that the activin A blocker follistatin enhances 1,25D3 stimulated mineralization (Woeckel et al., 2013). The above mentioned induction of carboxylated osteocalcin by 1,25D3 may fit this hypothesis on preventing over-mineralization. Accumulation of osteocalcin in the ECM of human osteoblast cultures stimulated by 1,25D3 is inhibited by warfarin (antagonist of vitamin K) while vitamin K2 (cofactor of γ-carboxylase) enhanced the 1,25 D3 effect (Koshihara and Hoshi, 1997). 1,25D3 stimulated mineralization was significantly augmented by warfarin (Woeckel et al., 2013). These data on activin A, follistatin, warfarin, and vitamin K put forward a 1,25D3 induced regulatory mechanism to guarantee optimal mineralization (Woeckel et al., 2013). Differences in these regulatory loops may also be part of the differences in 1,25D3 effects in human and murine osteoblast studies.

The most well-known mechanism to limit the biological activity of 1,25D3 is its degradation via 24-hydroxylation. 1,25D3 potently induces CYP24A1, which encodes for the enzyme 24-hydroxylase, in osteoblasts. 24-Hydroxylation is the first step in the degradation cascade of active 1,25D3 (Ohyama et al., 1994). However, hydroxylation at the C-24 position doesn't directly lead to an inactive vitamin D molecule. Henry and Norman demonstrated the significance of 24,25-dihydroxyvitamin D3 (24,25D3) for normal chicken egg hatchability and calcium and phosphorus homeostasis (Henry and Norman, 1978; Norman et al., 1980). Already in 1980 it was shown that 24,25D3 directly stimulates calcification of bone in interaction with PTH and that the number and size of resorption sites in bone is decreased by 24,25D3 (Endo et al., 1980; Galus et al., 1980). Several other studies supported a positive effect of 24,25D3 on bone metabolism (Matsumoto et al., 1985; Tam et al., 1986; Kato et al., 1998) while one study showed no effect of 24,25D3 on histomorphometric parameters in ovariectomized rats (Erben et al., 1992). Administration of 24,25D3 in combination with 1,25D3 improved fracture healing in chickens (Seo et al., 1997) and interestingly, 24,25D3 serum levels correlated to fracture healing (Seo and Norman, 1997). Studies with the CYP24A knockout mouse supported a role for 24,25D3 in fracture repair (St-Arnaud, 2010). Albeit in a human study no positive association with femoral fracture was observed (Weisman et al., 1978). However, a study in pre-dialysis renal insufficiency patients supported a direct, i.e., PTH-independent, functional role of 24,25D3 in bone (Birkenhäger-Frenkel et al., 1995). These data suggest a direct effect on osteoblasts. *In vitro* studies with human osteoblasts have shown that indeed 24,25D3 has direct effects similar to that of 1,25D3 (Van Driel et al., 2006b). A recent comparative gene expression profiling study of 1,25D3, 24,25D3, and 25D3 in primary human and mouse fibroblasts suggested induction of metabolite specific sets of genes and pathways (Tuohimaa et al., 2013). It is important to note that the fact whether biological active levels of 24,25D3 or 1,24,25-trihydroxyvitamin D3 (1,24,25D3) can be reached fully depends on the velocity of the subsequent steps in the degradation pathway after the initial 24-hydroxylation step.

We have shown that osteoblasts besides degradation of active 1,25D3, are able to convert 25-hydroxyvitamin D3 (25D3) into the biologically most active form 1,25D3, suggesting a direct relationship between 1,25D3 synthesis and bone (Van Driel et al., 2006a). This study showed functionality of 1α-hydroxylation in human osteoblast differentiation. 25D3 induced expression of CYP24, osteocalcin and stimulated ALPL activity and mineralization, which was blocked by inhibition of 1α-hydroxylase by ketoconazole. Downregulation of CYP27B1 in human osteoblasts or perturbation of CYP27B1 supported the requirement of 1α-hydroxylase for the effect on human MSC proliferation and osteogenic differentiation (Atkins et al., 2007; Geng et al., 2011a). CYP27B1 expression is reduced in MSC of older subjects and resistance to 25D3 induced osteoblast formation points to an aging effect (Geng et al., 2011b). The 1α-hydroxylase-dependent 25D3 stimulation of ALPL activity in human MSC was blocked by histone deacytylase inhibition (Zhou et al., 2013). Of interest, 25D3 has been shown to regulate gene expression in a gene expression profiling study with CYP27B1 deficient fibroblasts (Tuohimaa et al., 2013). This suggests that 25D3 may act independent of 1α-hydroxylation.

Up to now the data on 1,25D3 production by osteoblasts are derived from *in vitro* studies. *In vivo* significance of CYP27B1 and 1,25D3 formation in osteoblasts needs yet to be proven, for example by knocking out CYP27B1 specifically in osteoblasts. However, the observed discrepancies in effects on human-murine osteoblasts may hamper this approach. Although yet *in vivo* proof is lacking, the principal of local synthesis of 1,25D3 in bone may explain the observed associations of 25D3 and not of 1,25D3 with bone as well as other parameters (Hewison et al., 2004; Anderson et al., 2013). Besides CYP27B1, osteoblasts also express the receptors megalin and cubulin that are involved in cellular uptake of 25D3 via endocytosis of the vitamin D binding protein (DBP) (Van Driel et al., 2006a; Atkins et al., 2007). Linking back to the above discussed interaction between locally produced growth factors and 1,25D3 is the regulation of CYP27B1 in osteoblasts. Albeit 1,25D3 itself inhibits CYP27B1 expression in MSC as well as in the kidney (Zhou et al., 2010), the regulation appears to be different and more complex than in the kidney involving local regulators. Several locally in bone produced factors affects CYP27B1 expression: TGFβ suppresses 5′-flanking region of CYP27B1 (Turner et al., 2007) and interferon-β reduces while interleukin-1 and IGF-I increase CYP27B1 expression in mature human osteoblasts (Van Driel et al., 2006a; Zhou et al., 2010; Woeckel et al., 2012). The effect of interleukin-1 points to the involvement of NF-κB in stimulation of CYP27B1 expression in human osteoblasts. This is supported by the interferon-β inhibition of NF-κB in synoviocytes (Van Holten et al., 2004) and CYP27B1 regulation in human dendritic cells (Hewison et al., 2003).

1,25D3 plays an important role in maintaining bone health either via controlling calcium and phosphate homeostasis or via direct effects on osteoblasts. This latter is supported by the direct effects of 1,25D3 on osteoblast differentiation, expression and activity of bone formation related proteins and enzymes, and mineralization. The complete vitamin D endocrine system, from receptor to enzymes involved in 1,25D3 synthesis and breakdown, is present in the osteoblast, pointing to an autocrine/paracrine 1,25D3 function in bone. This is the more so interesting as over the past decade it has become clear that osteoblasts are not only involved in bone metabolism but that they also form the hematopoietic stem cell (HSC) niche controlling renewal of HSCs and differentiation of the immune cells (Calvi et al., 2003). Moreover, these HSC niches are also the sites of bone metastasis (Shiozawa et al., 2011). Considering the 1,25D3 effect on the immune system and tumor cell growth it is tempting to speculate that autocrine/paracrine action of 1,25D3 is also beyond bone metabolism and important for other regulatory functions of osteoblasts. It is therefore of critical importance to understand the full picture of 1,25D3 effects on osteoblasts. One of the approaches to obtain information on the effects of 1,25D3 on osteoblasts and MSC in an unbiased way is by omics approaches in combination with bioinformatics. In the next paragraph the current available 1,25D3 gene expression profiling studies of osteoblasts will be discussed.

## Vitamin D and gene transcription in the context of osteoblast differentiation and bone formation

1,25D3 has been shown to regulate the expression of various genes related to osteoblast proliferation and differentiation. BMP-2 induced bone formation has been suggested to be enhanced by 1,25D3 induced c-MYC expression (Piek et al., 2010). Induction of Insulin-like growth factor-binding proteins (IGFBP)-2, -3, and -4 expression by 1,25D3 in human MSC may play a role in stimulation of osteogenic differentiation (Kveiborg et al., 2001). Recently, Li and coworkers (Li et al., 2013) demonstrated that IGFBP-3 interacts with the VDR and negatively regulates CYP24 and BGLAP expression. Overexpression of IGFBP-3 inhibited the 1,25D3 activation of ALP in MG-63 human osteosarcoma cells.

1,25D3 also regulated Forkhead Box O (FoxO) transcription factors in murine MC3T3 osteoblasts with FoxO3a being up-regulated while FoxO1 was down-regulated, and FoxO4 not affected. Knockdown of the FoxO's didn't change 1,25D3 inhibition of cell growth but led to increased accumulation of reactive oxygen species after 1,25D3 treatment (Eelen et al., 2013). This may be linked to cellular metabolism and the high energy demanding process of bone formation (Komarova et al., 2000; Chen et al., 2008; Bruedigam et al., 2010). Unfortunately, the effect of FoxO's knockdown on mineralization in these murine MC3T3 osteoblast cultures was not reported. 1,25D3 increased vascular endothelial growth factor (VEGF) expression in human and rat osteoblasts is interesting considering the relationship between bone formation and angiogenesis (Wang et al., 1996; Schlaeppi et al., 1997; Corrado et al., 2013). VEGF has been shown to be involved in the 1,25D3 bone anabolic effect (Wang et al., 1997).

Recent studies placed miRNAs in the 1,25D3 mechanism of action spectrum in osteoblasts. Five miRNAs were found to be differentially expressed in primary human osteoblast after 6 h of treatment with 1,25D3 (Lisse et al., 2013a,b). Interestingly, miR-637 and miR-1228 are two miRNAs located intergenic in DAPK3 and LRP1, respectively. miR-1228 was upregulated and coexpressed with its host gene LRP1 suggesting a conventional VDRE- mediated transactivation upon 1,25D3 treatment. Since LRP1 is known to mediate the canonical Wnt pathway in fibroblasts (Terrand et al., 2009), this suggests an indirect regulation of Wnt signaling by 1,25D3 adding to other data on 1,25D3 and Wnt signaling interaction (Fretz et al., 2007; Haussler et al., 2010). The target of miR-1228, BMP2K, was previously identified to be increased in mouse osteoblasts upon treatment with BMP2 (Kearns et al., 2001). Stable expression of BMP2K in mouse osteoprogenitor cells decreased ALPL activity and osteocalcin mRNA levels. This suggests that 1,25D3 induced expression of miR-1228 may affect osteoblast differentiation via down-regulation of BMP2K.

On the contrary, 1,25D3 upregulated miR-637 while its host gene was downregulated suggesting a different way of regulation of the two transcripts. miR-637 stimulated the degradation of COL4A mRNA levels that is expressed in the basement membrane and is downregulated during early differentiation of mouse MC3T3-E1 osteoblasts (Hong et al., 2010). It is becoming evident that miRNAs play an important role in osteoblast differentiation and bone formation (Lian et al., 2012) and in the near future more data on their role in 1,25D3 action in osteoblasts will come forward (Lisse et al., 2013a).

## Identification of 1,25D3 target genes in osteoblasts

In the past various studies have investigated the effects of 1,25D3 on target gene expression and VDR binding to DNA response elements. Only a few of these genome-wide studies have investigated the effects of 1,25D3 in the context of osteoblasts (Table 1). The studies that carried out are very heterogenic with regard to the differentiation stage of the cells (MSC vs. primary osteoblasts vs. Cell line), time points of treatment (2–6 h after treatment) and the 1,25D3 concentration that is used (1–100 nM). Together this makes it difficult to compare the different studies. Systematic analyses of both mRNA gene expression profiling and VDR binding experiments at early time points after induction with 1,25D3 will uncover direct target genes. Below we will address a few of these studies and the results obtained.

**Table 1:** **Genome-wide studies of vitamin D and osteoblasts**.

**Publication**	**Experiment**	**Species**	**Cell type**	**Treatment**
Lisse et al., 2013a,b	Expression profiling miRNA	Homo sapiens	Primary osteoblasts	1,25D3 10^−8^ M 6 h
Woeckel et al., 2012	Expression profiling mRNA	Homo sapiens	Pre-osteoblasts svHFO	1,25D3 10^−8^ M 2 and 24 h
Tarroni et al., 2012	Expression profiling mRNA	Homo sapiens	Primary osteoblasts	1,25D3 10^−7^ M 24 h
Grundberg et al., 2011	Expression profiling mRNA	Homo sapiens	Trabecular bone	1,25D3 10^−7^ M 2 and 24 h
Piek et al., 2010	Expression profiling mRNA	Homo sapiens	MSCs	1,25D3 10^−8^ M 0, 1, 3, 6, 12, 24, 48, 72, 120, 192, and 288 h
Meyer et al., 2010	VDR localization ChIP-chip	Mus musculus	Pre-osteoblasts MC3T3-E1	1,25D3 10^−7^ M 3 h
Woeckel et al., 2010	Expression profiling mRNA	Homo sapiens	Pre-osteoblasts svHFO	1,25D3 10^−8^ M 3, 7, 12, and 19 days
Eelen et al., 2004	Expression profiling mRNA	Mus musculus	Pre-osteoblasts MC3T3-E1	1,25D3 10^−8^ M 6 and 12 h
Farach-Carson and Xu, 2002	Expression profiling mRNA	Rattus norvegicus	Osteosarcoma ROS 17/2.8	1,25D3 10^−9^ M 0, 6 and 24 h

*Database searches were performed using Bone[Title/Abstract] OR osteoblast[Title/Abstract] AND vitamin D AND microarray in Pubmed (http://www.ncbi.nlm.nih.gov/pubmed) and GEO (http://www.ncbi.nlm.nih.gov/geo/)*.

### ChIP analyses in osteoblasts

Upon binding of 1,25D3 to the VDR, the VDR binds with its heterodimeric partner retinoid X receptor (RXR) on the vitamin D receptor response elements (VDRE). The VDRE consists of the hexameric sequence AGGTCAxxxAGGTCA (Ozono et al., 1990) but variants to the conserved sequence have been identified (Meyer, 2005). Due to the diversity of VDRE, bioinformatics approaches are limited in identifying whole genome VDR binding sites. To identify direct target genes of VDR, genome-wide approaches such as ChIP-chip or ChIP-seq approaches have to be performed. Systematic analyses of VDR binding upon activation by 1,25D3 combined with bioinformatics approaches identifies VDRE (VDR response elements) and subsequently direct targets of Vitamin D signaling. A few studies have started to identify 1,25D3 target genes is various cell types such as a human derived lymphoblastoid cell line (Ramagopalan et al., 2010) and monocytes (Heikkinen et al., 2011). Recently, the first VDR binding experiments in osteoblasts were published (Meyer et al., 2010). Meyer et al. analyzed the genomic locations that bind VDR, RXR, RNA polymerase II and acetylated H4 after 3 h treatment with 1,25D3 in mouse MCT3T-E1 osteoblasts. Interestingly, only 13% of the identified sites was located in classical promoter regions upstream vitamin D target genes. The majority of sites that were found to bind VDR, RXR and acetylated H4 were located distal (43%) and within intronic and exonic regions (44%). This demonstrates that distal transcriptional control contributes to the majority of vitamin D3-mediated transcription. Genome wide ChIP-seq analyses with human osteoblasts should illustrate whether binding of VDR at distal locations is conserved.

Pilot analysis of our gene expression profiles of osteogenic and adipogenic MSCs illustrated that many known 1,25D3 responsive genes (on basis of Ingenuity database; www.ingenuity.com) are dynamically expressed during adipogenic as well as osteogenic differentiation (data not published). This data does not directly show that these genes are regulated by 1,25D3 but it suggests that 1,25D3-responsive genes can have a role during the differentiation of mesenchymal precursors. Many of the two-fold regulated genes during osteogenic differentiation and those that were identified previously to be regulated by 1,25D3 are involved in Cell Cycle (41/162; GO:0007049), response to steroid hormone (21/162; GO:0048545), regulation of phosphate metabolic process (26/162, GO:0019220), regulation of apoptosis (31/162, GO:0042981), extracellular region part (36/162; GO:0044421). ChIP analyses using VDR and expression profiling of 1,25D3 transcriptional activity against the backdrop of osteogenic MSC will be needed to demonstrate the importance of VDR—1,25D3 binding in osteoblast function either in bone formation, regulation of osteoclast formation and activity or in the stem cell niche.

### Gene expression profiling in osteoblasts

Besides binding of the VDR to gene regulatory elements, important information on the effect of 1,25D3 on osteoblasts comes from expression profiling studies upon 1,25D3 treatment. Several gene expression profiling studies have been performed to examine the effect of 1,25D3 on RNA expression in osteoblasts. Gene expression profiling in murine MC3T3 cells showed down-regulation of DNA replication genes (Eelen et al., 2004) which fits the earlier observed inhibition of proliferation in these cells. Gene profiling of 1,25D3 treated human osteoblasts at multiple days during the differentiation phase before mineralization did not show regulation a specific set of DNA replication genes (Woeckel et al., 2010). Cell death, RNA splicing translation, and cell cycle genes were identified by Gene Ontology analyses as being most significantly overrepresented (Woeckel et al., 2010). Only 0.6 % (3 genes) of the genes changed in expression during the mineralizing period were also changed prior to mineralization (Woeckel et al., 2010). This study demonstrated that 1,25D3 has different effects on gene expression dependent on the differentiation stage of the cells and should be carefully addressed when investigating the effects of 1,25D3 on mesenchymal stem/stromal cells and differentiated osteoblasts.

Tarroni et al. found that upon 24 h treatment of human osteoblasts with 1,25D3 most genes were upregulated (136 up vs. 20 down) indicating the transcriptional activation of 1,25D3 (Tarroni et al., 2012). Pathway analyses identified various biological functions and/or diseases related to bone metabolism and cellular processes/molecular functions related to skeletal development. The link with skeletal development is supported by another study showing 1,25D3 induced expression in human and mouse osteoblasts of the odd-skipped related genes Osr1 and Osr2, known from expression in the developing limb (Verlinden et al., 2013).

Tarroni et al. also showed strong change in expression of genes linked to inflammation or immune and lymphatic system development (Tarroni et al., 2012). In line with this, is the observation of a gene profiling study showing interferon-related genes being overrepresented after 1,25D3 treatment of human osteoblasts. The interferon signaling related genes were down-regulated by 1,25D3 (Woeckel et al., 2012). The observations on processes related to the immune system are interesting from at least two points of view. Firstly, because of the link between the immune system and bone and the effect of immune cells-derived cytokines on bone metabolism, e.g., in conditions like rheumatoid arthritis. Secondly, considering the above mentioned role of the osteoblasts in the stem cell niche and control of hematopoietic stem cell renewal and differentiation. The expression profiling data and the identification of functions and processes related to the immune system may support a role of vitamin D in osteoblasts control the stem cell niche (Kawamori et al., 2010).

## Conclusion

Vitamin D can regulate bone metabolism in an indirect way via controlling calcium and phosphate homeostasis but also via direct effects on osteoblasts. In fact, the complete vitamin D endocrine system is present in osteoblasts. This enables osteoblasts to respond not only to vitamin D via the VDR but also to synthesize the biological most active vitamin D metabolite 1,25D3 and to act in an autocrine/paracrine manner. Vitamin D directly regulates gene expression and stimulates mineralization in *ex vivo* cultures of human and rat osteoblasts. The effect on mineralization may depend on species and/or environmental context that can alter the eventual vitamin D effect. Besides effects on bone metabolism, vitamin D effects on osteoblasts may be related to additional functions of osteoblasts such as the hematopoietic stem cell niche. Interesting in this respect is that gene expression profiling studies on vitamin D-treated osteoblasts revealed genes and processes related to the immune system. Further studies are needed to delineate these non-bone metabolism related effects of vitamin D in osteoblasts in greater detail at cellular and molecular level. A future challenge will be to construct networks representing the effects of vitamin D, either in bone metabolism- or in non-bone metabolism-related processes, against the backdrop of osteoblast differentiation by systems biological approaches.

### Conflict of interest statement

The authors declare that the research was conducted in the absence of any commercial or financial relationships that could be construed as a potential conflict of interest.
